# Water extract of brewers’ rice induces apoptosis in human colorectal cancer cells via activation of caspase-3 and caspase-8 and downregulates the Wnt/β-catenin downstream signaling pathway in brewers’ rice-treated rats with azoxymethane-induced colon carcinogenesis

**DOI:** 10.1186/s12906-015-0730-4

**Published:** 2015-06-30

**Authors:** Bee Ling Tan, Mohd Esa Norhaizan, Ky Huynh, Sulaiman Rahman Heshu, Swee Keong Yeap, Hamzah Hazilawati, Karim Roselina

**Affiliations:** Department of Nutrition and Dietetics, Faculty of Medicine and Health Sciences, Universiti Putra Malaysia, 43400 Serdang, Selangor Malaysia; Research Centre of Excellent, Nutrition and Non-Communicable Diseases (NNCD), Faculty of Medicine and Health Sciences, Universiti Putra Malaysia, 43400 Serdang, Selangor Malaysia; Laboratory of Molecular Biomedicine, Institute of Bioscience, Universiti Putra Malaysia, 43400 Serdang, Selangor Malaysia; Department of Cell and Molecular Biology, Faculty of Biotechnology and Biomolecular Sciences, Universiti Putra Malaysia, 43400 Serdang, Selangor Malaysia; Department of Veterinary Pathology and Microbiology, Faculty of Veterinary Medicine, Universiti Putra Malaysia, 43400 Serdang, Selangor Malaysia; Department of Food Technology, Faculty of Food Science and Technology, Universiti Putra Malaysia, 43400 Serdang, Selangor Malaysia

**Keywords:** Brewers’ rice, Colorectal cancer, Wnt signaling, Cyclin D1, c-myc

## Abstract

**Background:**

Brewers’ rice, is locally known as *temukut*, is a mixture of broken rice, rice bran, and rice germ. The current study is an extension of our previous work, which demonstrated that water extract of brewers’ rice (WBR) induced apoptosis in human colorectal cancer (HT-29) cells. We also identified that brewers’ rice was effective in reducing the tumor incidence and multiplicity in azoxymethane (AOM)-injected colon cancer rats. Our present study was designed to identify whether WBR confers an inhibitory effect via the regulation of upstream components in the Wnt signaling pathway in HT-29 cells. To further determine whether the *in vitro* mechanisms of action observed in the HT-29 cells inhibit the downstream signaling target of the Wnt/β-catenin pathway, we evaluated the mechanistic action of brewers’ rice in regulating the expressions and key protein markers during colon carcinogenesis in male Sprague–Dawley rats.

**Methods:**

The mRNA levels of several upstream-related genes in the Wnt signaling pathway in HT-29 cells treated with WBR were determined by quantitative real-time PCR analyses. Caspase-3 and −8 were evaluated using a colorimetric assay. Male Sprague–Dawley rats were administered two intraperitoneal injections of AOM in saline (15 mg/kg body weight) over a two-week period and received with 10, 20, and 40 % (w/w) brewers’ rice. The expressions and protein levels of cyclin D1 and c-myc were evaluated by immunohistochemical staining and western blotting, respectively.

**Results:**

The overall analyses revealed that the treatment of HT-29 cells with WBR inhibited Wnt signaling activity through upregulation of the casein kinase 1 (*CK1*) and adenomatous polyposis coli (*APC*) mRNA levels. We discovered that the treatment of HT-29 cells with WBR resulted in the induction of apoptosis by the significant activation of caspase-3 and −8 activities compared with the control (*P* < 0.05). *In vivo* analyses indicated that brewers’ rice diminished the β-catenin, cyclin D1, and c-myc protein levels.

**Conclusions:**

We provide evidence that brewers’ rice can induce apoptosis and inhibit the proliferation of HT-29 cells through regulation of caspase-dependent pathways and inhibit the Wnt/β-catenin downstream signaling pathway *in vivo*. We suggest that brewers’ rice may be a useful dietary agent for colorectal cancer.

## Background

Colorectal cancer has become a global health problem, represents the third most common cancer after lung and breast cancers and contributes for nearly 10 % of the total cases of cancer and approximately 8 % of the total cancer deaths [1]. Chemoprevention using natural products is considered a practical strategy for reducing the ever-increasing cancer incidence [2]. The intervention of multistage carcinogenesis through the modulation of intracellular signaling pathways may provide the molecular basis of chemoprevention.

Emerging evidence reveals that Wnt/β-catenin signaling plays critical roles in human biology [3]. Inappropriate stimulation of Wnt pathway is linked with many cancers [4]. The amount of cytoplasmic β-catenin is maintained at a low amount via ubiquitin-proteasome-mediated phosphorylation and is regulated by a destruction complex contained of AXIN1, adenomatous polyposis coli (APC), casein kinase 1 (CK1), and glycogen synthase kinase 3β (GSK3β) [5]. Cyclin D1 is a Wnt target gene [6], and mutations of this signaling pathway contribute to nearly 90 % of colorectal cancer [7]. Gene mutation in the Wnt pathway, including inactivating mutations of the *APC* gene and stimulating mutations in β-catenin, lead to the nuclear accumulation of β-catenin and subsequently result in the formation of a complex with T-cell factor (TCF)/lymphoid enhancing factor (LEF) to stimulate gene transcription [8]. The TCF/LEF binding sites on promoters of cell proliferation genes, including cyclin D1 and c-myc [6], result in the stimulation of aberrant mutations in the tumorigenic signals of the colonic crypts.

Rice is one of the main staple foods in approximately half of the world’s population [9]. The milling of paddy rice results in an approximately 70 % yield of rice as its major product, with some of the unconsumed portions of the rice produced include rice husk (20 %), rice bran (8 %), and rice germ (2 %) [10, 11]. Brewers’ rice, which is known locally as *temukut*, is typically used as an animal feed and brewing material [12]. Several studies as reported by Esa et al. [13] have demonstrated that rice by-products can inhibit human colon cancer cell proliferation and reduce the AOM-induced colon tumors in rats. Therefore, the purpose of this study was to investigate whether water extract of brewers’ rice (WBR) confers an inhibitory effect via the regulation of upstream components in the Wnt signaling pathway in colorectal cancer (HT-29) cells. To further determine whether the *in vitro* mechanisms of action observed in HT-29 cells inhibit the downstream signaling target of the Wnt/β-catenin pathway, we evaluated the mechanistic action of brewers’ rice in regulating the expressions and key protein markers during colon tumorigenesis in male Sprague–Dawley rats.

We observed that the treatment of HT-29 cells with WBR inhibited Wnt signaling activity through upregulation of the *CK1* and *APC* mRNA levels. In addition, the treatment of HT-29 cells with WBR caused in the induction of apoptosis by the significant activation of caspase-3 and −8 activities compared with the control (*P* < 0.05). *In vivo* analyses also showed that brewers’ rice diminished the β-catenin, cyclin D1, and c-myc protein levels.

## Materials and methods

### Chemicals and reagents

Azoxymethane (AOM), 10 % (v/v) neutral buffered formalin, TRI Reagent®, and specific primers were bought from Sigma (St. Louis, MO, USA). Dulbecco’s Modified Eagle Medium (DMEM), RPMI-1640 medium, Mycoplex™ fetal bovine serum (FBS), penicillin and streptomycin (100×), and trypsin EDTA (1×) were obtained from PAA Laboratories GmBH (Pasching, Austria). Colorimetric assay kit for caspase was bought from Genscript Corporation Inc (Piscataway, NJ, USA). High capacity RNA-to-cDNA Kit and SYBR® Select Master Mix (CFX) were purchased from Applied Biosystems (Foster City, CA, USA). Western blotting reagents were purchased from Bio-Rad (Hercules, CA, USA). All other chemicals and reagents used were of analytical grade and bought from Sigma-Aldrich (St. Louis, MO, USA).

### Stabilization of brewers’ rice

Freshly milled brewers’ rice samples (MR 219) were obtained from BERNAS Milling Plant (Seri Tiram Jaya, Selangor, Malaysia). Stabilized samples were carried out as previously described by Tan et al. [14]. Brewers’ rice consists of broken rice (95.16 ± 4.62 %), rice bran (3.60 ± 0.39 %), and rice germ (1.11 ± 0.07 %), while WBR is the extract of brewers’ rice. WBR contains the bioactive compounds from these three major compositions in brewers’ rice (broken rice, rice bran, and rice germ).

### Preparation of water extract of brewers’ rice

Stabilized brewers’ rice was extracted with water following Yu et al. [15].

### Cell culture and treatment

The human colorectal cancer (HT-29) cells were bought from American Type Culture Collection (ATCC; Rockville, MD, USA), and the cells were cultured in DMEM supplemented with 10 % (v/v) FBS, 100 IU/mL penicillin, and 100 μg/mL streptomycin. HT-29 cells were maintained and incubated at 37 °C in a humidified atmosphere with 5 % CO_2_ atmosphere. The cells were treated with three different concentrations of WBR (16, 32, and 64 μg/mL) for 72 h.

### Gene expression

The TRI Reagent® was used to isolate the total RNA, according to the manufacturer’s protocol. Two micrograms of RNA per 20 μL was reverse-transcribed using the High-Capacity RNA-to-cDNA Kit according to the manufacturer’s protocol. The reverse-transcription reaction was performed using an Authorized Thermal Cycler (Eppendorf, NY, USA). The cDNA was then ready for use as a template for amplifications through real-time PCR reactions. Quantitative real-time PCR was carried out using SYBR® Select Master Mix (CFX). The nucleotide primer sequences originating from human cell lines are shown in Table 1. All of the samples and controls were determined in triplicate using the BioRAD-iQ™ 5 Multicolor Real-Time PCR Detection System (Hercules, CA, USA). The quantitative real-time PCR data were analyzed using the CFX Manager™ software (version 1.6, Bio-Rad, Hercules, CA, USA). Beta-actin (*ACTB*), glyceraldehyde-3-phosphate dehydrogenase (*GAPDH*), and 18S rRNA were used as housekeeping genes to normalize the target genes expression.

**Table 1:** Nucleotide sequence of PCR primers for amplification and sequence-specific detection of cDNA (obtained from GenBank database)

Primer name [accession number]	Oligonucleotides (5′-3′)
	Sequence
*AXIN1* [XM_005255610.2]	F- TTTCACCGAAGATGCTCCCC
	R- CACTGCCCTCAGGCTCATAC
*CK1* [NM_001271741.1]	F- GAGATCCCTTTCCCAGAGTGC
	R- TTTGTGAAGGGCTTCTCGGC
*GSK3β* [BC012760]	F- CGAATGGGGAACAGTCGAGG
	R- TCGGAAATGCGACGGGAAAC
*APC* [NM_000038.5]	F- AGCAAGTTGAGGCACTGAAGA
	R- TCCCGGCTTCCATAAGAACG
*LRP6* [NM_002336.2]	F- ACATGACAGGTCGAGAGGGT
	R- CCAAGCCACAGGGATACAGT
*ACTB* ^a^ [NM_001101.3]	F- AGAGCTACGAGCTGCCTGAC
	R- AGCACTGTGTTGGCGTACAG
*GAPDH* ^a^ [NM_002046.4]	F- GGATTTGGTCGTATTGGGC
	R- TGGAAGATGGTGATGGGATT
18S rRNA^a^ [HQ387008.1]	F- GTAACCCGTTGAACCCCATT
	R-CCATCCAATCGGTAGTAGCG

*ACTB*- beta-actin, *APC*- adenomatous polyposis coli, *CK1*- casein kinase 1, *GAPDH*- glyceraldehyde-3-phosphate dehydrogenase, *GSK3β*- glycogen synthase kinase 3β, *LRP6*- low density lipoprotein receptor-related protein 6

^a^Housekeeping gene

### Caspase-3 and caspase-8 assay

The caspase-3 and −8 activities were assessed spectrophotometrically using a commercial colorimetric assay kit, based on spectrophotometric detection of the chromophore *p*nitroanilide (*p*NA) after cleavage from the specific substrates DEVD-*p*NA (for caspase-3) and IETD-*p*NA (for caspase-8). Initially, HT-29 cells were treated with three different concentrations (16, 32, and 64 μg/mL) of WBR for 72 h. The cells were centrifuged at 370 × g for 5 min to remove the medium and washed twice with phosphate-buffered saline (PBS) (pH 7.4). The cell pellets were then lysed with 50 μL of cold lysis buffer, followed by 1 h incubation period on ice. The resulting cell lysates were centrifuged at 9300 × g and 4 °C for 1 min, and the supernatants were collected. The protein concentration was quantified using a Bradford protein assay kit, according to the manufacturer’s protocols. Fifty μL of 2 × reaction buffers were mixed with 50 μL supernatant containing 200 μg of proteins in each tube, followed by 5 μL of caspase-3 or caspase-8 substrate. The suspension was collected and transferred to a 96-well microtiter plate and incubated at 37 °C for 4 h before measured at 405 nm using ELISA microplate reader.

### Animals, diet, and *in vivo* experimental procedures

The animal use protocol was approved by the Animal Care and Use Committee (ACUC) of the Faculty of Medicine and Health Sciences, Universiti Putra Malaysia (UPM) Serdang, Selangor (Reference number: UPM/FPSK/PADS/BR-UUH/00461). A total of 30 four-week-old male Sprague–Dawley rats were housed in plastic cages (two rats per cage) and maintained in an animal facility at a controlled temperature of approximately 25–27 °C and a relative humidity of 50 ± 10 % with 12-hour light/dark cycles. Hygienic condition was maintained by weekly changes of the woodchip beds. The animals were acclimatized for seven days prior to receiving the following defined diets for twenty weeks (*n* = 6 rats for each group): (G1), normal; (G2), AOM alone; (G3), AOM + 10 % (w/w) brewers’ rice; (G4), AOM + 20 % (w/w) brewers’ rice; and (G5), AOM + 40 % (w/w) brewers’ rice. During the acclimatization period, the animals were fed an American Institute of Nutrition (AIN)-93G diet and tap water *ad libitum*. Beginning at six weeks of age, the rats in G2-G5 were received intraperitoneal injections of azoxymethane (AOM) at a dose of 15 mg/kg body weight once weekly over a 2-week period. The rats in the normal group (G1) received an equal volume of normal saline (vehicle control). The animals were administered fresh food and tap water every day. The experimental diets were prepared weekly and kept at 4 °C. The components of an AIN-93G diet (Table 2) were adjusted based on the nutrient composition of brewers’ rice as previously described by Tan et al. [16].

**Table 2:** Composition of experimental diets

Ingredients (g/1000 g diet)	Group				
	G1	G2	G3	G4	G5
Brewers’ rice	-	-	100.0	200.0	400.0
Corn starch	397.5	397.5	315.3	233.2	68.9
Casein	200.0	200.0	191.0	182.0	164.0
Maltodextrin	132.0	132.0	132.0	132.0	132.0
Sucrose	100.0	100.0	100.0	100.0	100.0
Soybean oil	70.0	70.0	68.1	66.1	62.2
Powdered cellulose	50.0	50.0	44.7	39.4	28.7
AIN-93G mineral mix	35.0	35.0	33.4	31.9	28.8
AIN-93G vitamin mix	10.0	10.0	10.0	10.0	10.0
L-cystine	3.0	3.0	3.0	3.0	3.0
Choline bitartrate	2.5	2.5	2.5	2.5	2.5
tert-butylhydroquinone	0.014	0.014	0.014	0.014	0.014

G1 and G2, AIN-93G diet; G3, AIN-93G diet containing 10 % (w/w) of brewers’ rice; G4, AIN-93G diet containing 20 % (w/w) of brewers’ rice; G5, AIN-93G diet containing 40 % (w/w) of brewers’ rice

### Tissue sample collection

The animals were sacrificed after 20 weeks of treatment by anesthesia with diethyl ether. The whole colon tissues were removed, rinsed with PBS, opened and cut longitudinally, and fixed in 10 % (v/v) neutral buffered formalin. The colon tissue was processed for further analyses.

### Immunohistochemical staining of cyclin D1 and c-myc antigen

The colonic tissue sections were dehydrated, embedded in paraffin, and sectioned at 4-6-μm thickness. The detailed analyses for immunohistochemical evaluation were described previously [16]. The primary antibodies against cyclin D1 (diluted 1:100) and c-myc (diluted 1:50) (Abcam, UK) were incubated with the sections. The whole colon was divided into three sections, including distal, middle, and proximal colon. The scoring was done in three sections for each rat for both normal colon mucosa and the lesion (tumor). Cyclin D1 and c-myc localization were evaluated, recording whether the staining was localized at the cytoplasm (the cell was classified as positive at the cytoplasm) or at the nucleus (positive at the nucleus). A semi-quantitative scoring system was performed following Kohno et al. [17] to examine the antibody staining against cyclin D1 and c-myc. The total scoring was evaluated after summation of the extent and intensity of the staining (score = extent + intensity) in seven randomly selected fields from each section of the colon of each rat stained with the respective antibody under 100× magnification. Quantification of cyclin D1 and c-myc staining that represents the extent (number of stained (positive) cells) and the intensity of the staining across 21 fields for each rat. The percentages of positive cells were evaluated using the following scale: 0 = no stained cells in any field; 1 = positive staining in 1 to 25 % of the cells; 2 = positive staining in 26 to 50 % of the cells; 3 = positive staining in 51 to 75 % of the cells; and 4 = positive staining in 76 to 100 % of the cells. The strength of the staining intensity was examined using the following range: 0, no cell staining; 1+, mild staining; 2+, moderate staining; and 3+, strong staining. Hence, after summation, the maximum score was 7, and the minimum score was 0.

### Western blotting analysis

The proteins from rat colon tissue (100 mg) were extracted with 300 μL of radio immune precipitation assay (RIPA) lysis buffer and 3 μL of protease inhibitor cocktail. The colon tissue was homogenized for 30 s prior to incubation at 4 °C for 1 h with agitation. The protein concentration was quantified using a Bradford protein assay kit according to the manufacturer’s protocol using bovine serum albumin as the standard. The protein suspensions were aliquoted into PCR tubes and stored at −80 °C prior to sodium dodecyl sulfate-polyacrylamide gel electrophoresis (SDS-PAGE) analysis. Equal amounts (50 μg) of protein were separated by electrophoresis with 1× running buffer (0.025 M Tris base, 0.192 M glycine, and 0.1 % SDS; pH 8.3). The protein lysates and protein ladder were loaded onto gels, and the gels were run at 75 V for 15 min and then at 120 V for 45 min. After the protein samples were run on the SDS-PAGE gel, the gel was removed from the glass plate and rinsed in 1× Towbins transfer buffer (25 mM Tris-base, 190 mM glycine, 20 % (v/v) methanol; pH 8.3) at room temperature for 10 min. Protein transfer from the SDS-PAGE gel to a PVDF membrane was achieved by running the transfer apparatus at 100 V and 400 mA for 2 h. The PVDF membrane was blocked with blocking buffer (5 % skim milk solution) at room temperature for 1 h. The membranes were washed three times with phosphate-buffered saline in 0.1 % Tween-20 (PBST) for 10 min each before incubation with specific primary antibodies in 5 % skim milk PBST. Rabbit monoclonal antibody (E247) to β-catenin, rabbit monoclonal antibody (EPR2241) to cyclin D1, and rabbit monoclonal antibody (Y69) to c-myc were used at a 1:10,000 dilution (Abcam, UK) overnight at 4 °C on a roller mixer. After overnight incubation with the primary antibodies, the membranes were washed three times with PBST for 10 min each before incubation with goat polyclonal secondary antibody to rabbit IgG conjugated to horseradish peroxidase (HRP) (Abcam, UK) at a 1:10,000 dilution at room temperature for 1 h. The protein band was developed and detected using a chemiluminescence blotting substrate kit (Clarity western ECL substrate), and a chemiluminescence imager (Bio-Rad, USA) was used to view the protein bands on the membranes. Beta-actin (Abcam, UK) was used as a loading control to confirm that the protein concentrations in all of the loaded samples were equal. The densitometric analyses of the band intensities obtained were performed using the Image J Software (National Institute of Health, NIH, Bethesda, MD, USA).

### Statistical analyses

The statistical data were expressed as the mean ± standard deviation (SD) and analyzed using a one-way analysis of variance (ANOVA). Statistical analyses were conducted following the Statistical Package for Social Science (SPSS) version 19.0 (SPSS Inc., Chicago, IL, USA). A P-value of < 0.05 was considered significant.

## Results and discussion

### WBR upregulates the mRNA level of *CK1* in HT-29 cells

**Fig. 1:**
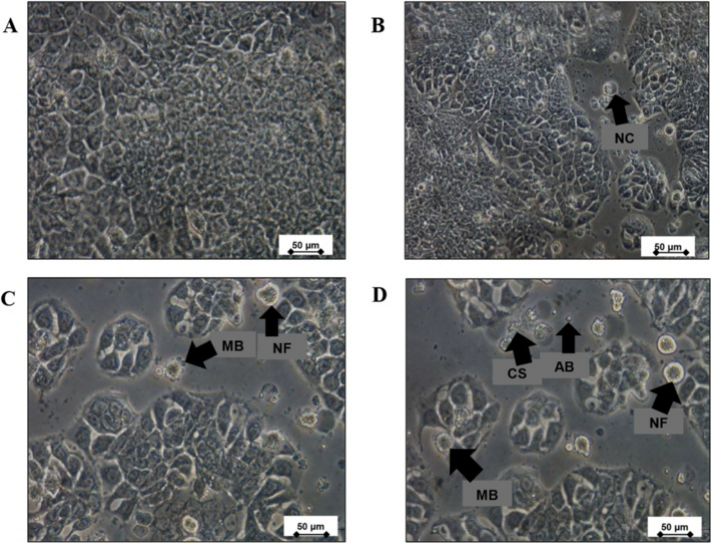
Morphological changes of HT-29 cells observed under an inverted light microscope. **a** Untreated HT-29 cells, HT-29 cells treated with water extract of brewers’ rice (WBR) at concentrations of **b** 16 μg/mL, **c** 32 μg/mL, and **d** 64 μg/mL. The cells showed typical characteristics of apoptosis, such as nuclear compaction (NC), apoptotic bodies (AB), membrane blebbing (MB), cellular shrinkage (CS), and nuclear fragmentation (NF) (magnification 400×). Scale bars in the figures indicate 50 μm (Source: Tan et al. [19])

According to published guidelines, any extract that exerts potentially cytotoxic effects should have an IC_50_ less than 100 μg/mL [18]. We previously reported that WBR and methanol extract of brewers’ rice (MBR) is cytotoxicity towards colorectal cancer (HT-29) cells with IC_50_ values of 38.33 ± 6.51 μg/mL and 54.00 ± 5.29 μg/mL, respectively [14], as evaluated through a 3-(4,5-dimethylthiazol-2-yl)-2,5-diphenyltetrazolium bromide (MTT) proliferation assay, whereas a lactate dehydrogenase (LDH) assay showed IC_50_ values of 21.88 ± 12.43 μg/mL and 34.50 ± 5.92 μg/mL for WBR and MBR, respectively [19]. The results showed that both WBR and MBR are sensitive to HT-29 cells, indicating that WBR and MBR can inhibit the proliferation of HT-29 cells. However, HT-29 cell line was more sensitive to WBR because the median inhibition concentration values were lower than that of MBR, as confirmed by MTT proliferation and LDH assays. The proliferation of HT-29 cells after treated with WBR is shown in Fig. 1. Therefore, we tested whether WBR can affect the Wnt signaling pathway in HT-29 cells.

To obtain further insights into the growth inhibitory effects of WBR in HT-29 cells are caused by the mechanistic action of WBR, the mRNA levels of several upstream-related genes in the Wnt signaling pathway were subsequently determined by quantitative real-time PCR analyses. In general, the Wnt/β-catenin signaling activity depends on the amount of β-catenin in the cytoplasm, and β-catenin is targeted for degradation by a destruction complex consisting of AXIN1, APC, CK1, and GSK3β [5]. Thus, the expression levels of *AXIN1*, *CK1*, *GSK3β*, and *APC* in response to WBR were examined using quantitative real-time PCR. Furthermore, to understand how WBR regulates the Wnt signaling, Wnt coreceptor low-density lipoprotein receptor-related protein 6 (*LRP6*), which is essential in signal transduction and is more likely to stimulate Wnt signaling when overexpressed, was analyzed. Given the broad cytotoxicity range of WBR against HT-29 cell line determined by the MTT and LDH assays, as reported in our earlier study [14, 19], only three concentrations (16, 32, and 64 μg/mL) were selected.

**Fig. 2:**
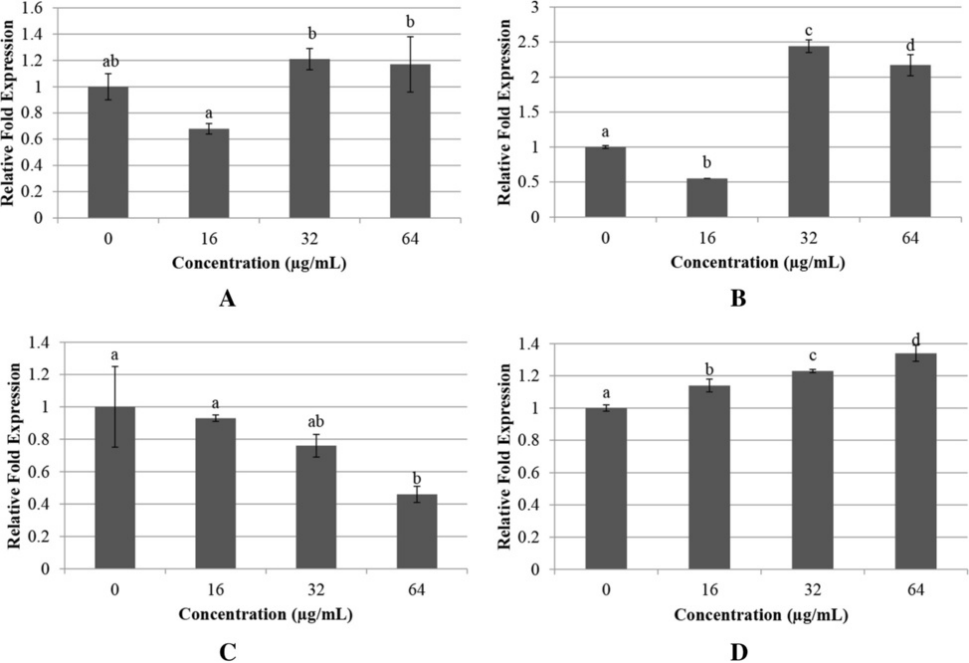
mRNA levels of HT-29 cells treated with WBR. Expression of **a** *AXIN1*, **b** casein kinase 1 (*CK1*), **c** glycogen synthase kinase 3β (*GSK3β*), and **d** adenomatous polyposis coli (*APC*) in Wnt signaling at mRNA levels in HT-29 cells incubated with WBR (*n* = 3). Value with different superscript letter indicates significant difference between groups by Tukey’s test (*P* < 0.05). *AXIN1* gene did not significantly differ between the control cells and those from the groups treated with 32 or 64 μg/mL WBR (*P* > 0.05). *CK1* mRNA level in the groups treated with 32 and 64 μg/mL WBR was significantly increased compared with the control (*P* < 0.05). *GSK3β* mRNA level in the group treated with 64 μg/mL WBR was significant decreased compared with the control (*P* < 0.05). *APC* gene in the groups treated with WBR was significantly elevated compared with the control (*P* < 0.05)

The stabilization of β-catenin through inhibition of destruction complex formation in Wnt signaling has been proposed by Cselenyi et al. [20]. AXIN1 overexpression induces the phosphorylation of β-catenin in cells that express truncated *APC* [21]. It has been reported that AXIN1 expression in SW480 cells stimulates the phosphorylation of β-catenin [22]. Therefore, it is plausible that the *AXIN1* mRNA level needs to be modulated to ensure proper Wnt signaling pathway. In the present study, we found that no significant difference in *AXIN1* gene between the control cells and the cells treated with 32 μg/mL WBR and between the control cells and the cells treated with 64 μg/mL WBR (*P* > 0.05) (Fig. 2a). Therefore, *AXIN1* mRNA level may not play a critical role in negatively regulating the Wnt signaling pathway to induce the phosphorylation and degradation of β-catenin.

In addition to the effects observed on *AXIN1*, β-catenin is targeted for phosphorylation, in part via the regulation by *CK1*γ. Beta-catenin is targeted for degradation via ubiquitin-mediated proteolysis through the action of CK1α [23]. As shown in Fig. 2b, the *CK1* mRNA level in HT-29 cells treated with 32 and 64 μg/mL WBR for 72 h was significantly increased compared with the control (*P* < 0.05). Thus, this finding suggested that treatment with WBR at concentrations of 32 and 64 μg/mL elevated the *CK1* mRNA level, and this increase may be associated with the induction of apoptosis, which may result to the phosphorylation of β-catenin in a Wnt pathway. Because WBR promotes the *CK1* mRNA level, the susceptibility of HT-29 cells to WBR may also be due to *GSK3β*. A previous study found that GSK3β may promote cancer development and tumorigenesis [24]. Thus, the effect of *GSK3β* in response to WBR was analyzed in HT-29 cells.

### Treatment with WBR inhibits the mRNA level of *GSK3β* in HT-29 cells

Because GSK3β modulates many substrates and signaling pathways, the mechanisms underlying its tumor suppressor or promoter activities are complex [25]. One of the critical roles of GSK3β on neoplastic transformation tumor development is more likely via the modulation of Wnt/β-catenin signaling. Active GSK3β induces the phosphorylation of β-catenin via ubiquitin-mediated proteasomal degradation, and the cytoplasmic β-catenin level thus remains low.

Our data revealed that *GSK3β* mRNA level was mainly present in untreated HT-29 cells (Fig. 2c). These findings were consistent with the results reported by Shakoori et al. [26], who found high levels of GSK3β in colon cancer cells and colorectal cancer patients compared with the normal counterparts. It is recognized that tumors depend on anaerobic pathways to shift glucose to ATP in the presence of ample oxygen, and tumor cells maintain the production of ATP via elevation of glucose influx to fuel the energy requirements for uncontrolled proliferation [27]. In the control (untreated HT-29 cells) group, the high *GSK3β* level observed in HT-29 cells may subsequently contribute to the high consumption of glucose by cancer cells via the inhibition of glycogen synthesis. Importantly, this finding indicates that high *GSK3β* levels may contribute to colorectal cancer, which contrasts its predicted role [28] as a tumor suppressor.

However, the *GSK3β* mRNA level in WBR-treated HT-29 cells was reduced, with a maximum reduction observed at a concentration of 64 μg/mL (Fig. 2c). These findings were further supported by Ougolkov et al. [29] and Cao et al. [30], who found that the suppression of GSK3β reduces pancreatic cancer cell survival and proliferation, suppresses the growth of ovarian cancer cells *in vitro*, and inhibits the formation of tumors in nude mice. Furthermore, non-steroidal anti-inflammatory drugs (NSAIDs), which are cancer chemopreventive agents, have also been reported to increase the phosphorylation of GSK3β and thereby induce the downregulation of β-catenin/TCF signaling [31]. In contrast, no significant differences were found in *GSK3β* between the control group and those from the groups treated with 16 μg/mL WBR or 32 μg/mL WBR (*P* > 0.05) (Fig. 2c). Taken together, these results suggested that treatment with WBR downregulated GSK3β via mechanisms other than Wnt/β-catenin signaling. Because we observed the upregulation of *CK1* and the downregulation of *GSK3β*, we further investigated the mRNA level of *APC*.

### Treatment with WBR upregulates the mRNA level of *APC* in HT-29 cells

APC is a large protein that binds to both β-catenin and AXIN1. It plays an essential role as a destruction complex in colorectal cancer [32]. As shown in Fig. 2d, the untreated cells (control) presented the lowest *APC* mRNA level compared with HT-29 cells treated with WBR. A previous study reported that most of the colorectal cancer cases present inactivating APC mutations [33]. Mutations in *APC* gene are observed in 70 % of colorectal cancer cases and are regarded as an initiating event due to an inability of the destruction complex to stimulate the degradation of β-catenin [34, 35]. When the destruction complexes contained of AXIN1, APC, CK1, and GSK3β are inhibited, subsequently resulted in the accumulation of cytoplasmic β-catenin [36] in the mutated HT-29 cell line. Our current data revealed that treatment with various concentrations (16, 32, and 64 μg/mL) of WBR increase the *APC* mRNA level in a dose-dependent manner. This finding indicated that the *APC* gene in HT-29 cells was significantly elevated after 72 h of incubation with WBR compared with the untreated HT-29 cells (control) (*P* < 0.05) (Fig. 2d).

The interaction of a Wnt ligand with transmembrane co-receptors, LRP6, suppresses the phosphorylation of the transcriptional coactivator β-catenin, which allows the translocation of β-catenin to the nucleus and activates the Wnt target genes [20]. Thus, the expression of *LRP6* in response of HT-29 cells was investigated to determine whether WBR can modulate *LRP6* at the mRNA level.

### Treatment with WBR downregulates the mRNA level of *LRP6* in HT-29 cells

The LRP6 is a crucial co-receptor in Wnt signaling [37]. Wnt ligands stabilize cytoplasmic β-catenin through the binding of the seven-transmembrane-domain receptor frizzled (Fz) and LRP5/LRP6 [37]. It has been reported that LRP6 is more potent than LRP5 in certain analyses; however, the qualitative differences in their mechanisms of action remain to be fully defined [38].

**Fig. 3:**
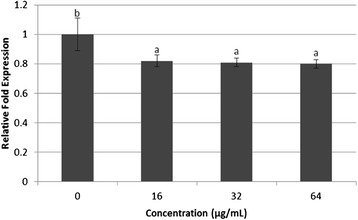
mRNA levels of HT-29 cells treated with WBR. Expression of low density lipoprotein receptor-related protein 6 (*LRP6*) mRNA level in HT-29 cells incubated with water extract of brewers’ rice (*n* = 3). Value with different superscript letter indicates significant difference between groups by Tukey’s test (*P* < 0.05). WBR treatment significantly reduced the *LRP6* mRNA level compared with the control (*P* < 0.05)

In addition to the destruction complex consisting of *AXIN1*, *CK1*, *GSK3β*, and *APC*, our data revealed that treatment with various concentrations (16, 32, and 64 μg/mL) of WBR significantly reduced the mRNA level of *LRP6* in HT-29 cells in a dose-dependent manner compared to the untreated cells (*P* < 0.05) (Fig. 3). Ligand-induced receptor downregulation has been shown to play a critical role in the regulation of the propagation and duration of growth factor receptor signaling and prevents stimulation of aberrant cell [39]. Collectively, these results suggest that the treatment of HT-29 cells with WBR results in the upregulation of *CK1* and *APC* and the downregulation of *GSK3β* and *LRP6* at the mRNA levels, which may be associated with a marked increase in apoptotic cell death, as reported in our previous study [19]. Apoptosis is another strategy used to eliminate cancerous cells [40]. It serves as a protective mechanism to destroy damaged cells prior to the manifestation of malignancy [41] without the activation of inflammatory responses [42]. To ascertain whether the growth inhibitory activity could be dependent on the activation of caspase-3 and caspase-8, which play vital roles in the modulation of apoptotic responses [43], the intracellular levels of caspase-3 and −8 in HT-29 cells after induction with WBR were investigated.

### Treatment with WBR promotes activation of caspase-3 and −8 activities in HT-29 cells

Resistance against apoptosis is important in survival and causes a drug resistance in cancer cells. Thus, apoptosis induction is a vital approach in the cancer chemoprevention and chemotherapy [2]. To gain a better understanding of the mechanism underlying apoptosis induction in WBR-treated HT-29 cells, we evaluated the action of WBR on caspase-3 and −8 activities. An apoptotic program is executed through a family of highly conserved cysteinyl aspartate-specific proteases known as caspases that dismantle the cell by cleaving high amounts of cellular substrates. Caspase-8 is considered an initiator caspase that is responsible for stimulating the downstream effector caspase-3. Modulating the mechanism through the activation of caspases is a vital molecular target in chemoprevention because these processes contribute to apoptosis [2].

**Fig. 4:**
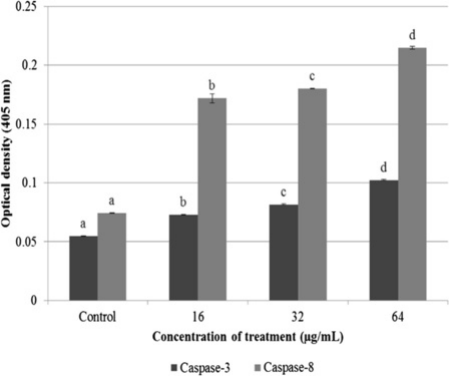
Caspase-3 and −8 activities in HT-29 cells treated with WBR (*n* = 3). Value with different superscript letter indicates significant difference between groups by Tukey’s test (*P* < 0.05). Both caspase-3 and −8 activities in the groups treated with WBR were significantly increased compared with the control (*P* < 0.05)

As shown in Fig. 4, WBR clearly increases the caspase-3 and −8 activities in a dose-dependent manner. The caspase-3 activity after treatment with 16, 32, and 64 μg/mL WBR was significantly increased compared with the control (*P* < 0.05). In addition to the upregulation of caspase-3, the caspase-8 activity in HT-29 cells was also significantly increased after treatment with WBR for 72 h (*P* < 0.05). Therefore, the findings may suggest that an increase in caspase-8 activity result in the stimulation of the downstream apoptotic executioner caspase-3, which subsequently activates the molecular cascade of apoptosis in HT-29 cells. Taken together, our data indicate that the caspase-3 and −8 activities are induced by WBR in a dose-dependent manner. WBR markedly increase in apoptosis, which was accompanied by the activation of caspase-3 and −8 activities. Collectively, these results demonstrated that the activation of caspase activities in HT-29 cells after treatment with WBR increments initiator caspase-8 and executioner caspase-3. Our data presented in this study demonstrated that WBR inhibits the proliferation of colon cancer *in vitro*, as reported in our earlier study [19], leading to programmed cell death, which was confirmed to be through the regulation of caspase-dependent pathways. To further verify whether the *in vitro* mechanisms of action observed in HT-29 cells could suppress the downstream signaling target of the Wnt/β-catenin pathway, we investigated whether brewers’ rice can reduce the expressions and key protein markers during colon carcinogenesis in male Sprague–Dawley rats using immunohistochemical and western blot evaluations. Thus, the levels of the downstream signaling targets cyclin D1 and c-myc in response to brewers’ rice were further evaluated in the colons of rats injected with AOM.

### Brewers’ rice downregulates the cyclin D1 and c-myc expression

The current study is an extension of our earlier work, which determined brewers’ rice to be an effective dietary agent for the reduction of colon tumor incidence and multiplicity formation in AOM-treated rats [16]. We also identified that brewers’ rice markedly reduces β-catenin expression in both the cytoplasm and the nucleus [16]. In the present study, male Sprague–Dawley rats received different doses (10, 20, and 40 % (w/w)) of brewers’ rice. A dosage of a preparation of 10 % (w/w) brewers’ rice was used as suggested by a previous study conducted by Boateng et al. [44] on rice bran and rice germ. This dosage has been reported to suppress tumor formation. In addition, higher concentrations (20 and 40 % (w/w) brewers’ rice) were also tested to determine the dose-dependent effect of brewers’ rice as a chemopreventive agent in a rat colon carcinogenesis experimental model. As shown in our previous study, feeding up to 40 % (w/w) brewers’ rice is well-tolerated and did not inhibit the growth of rats [16].

**Fig. 5:**
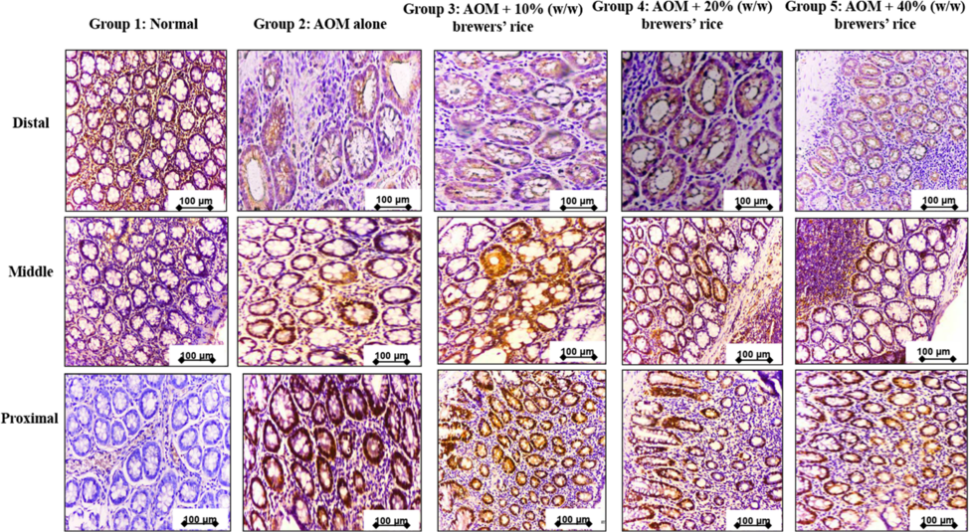
Immunohistochemical staining of cyclin D1. The expression of cyclin D1 in three sections, including distal, middle, and proximal colon in normal (Group 1), AOM alone (untreated) (Group 2) compared to groups of rats treated with 10 % (Group 3), 20 % (Group 4), and 40 % (Group 5) (w/w) of brewers’ rice (*n* = 3). Expression of cyclin D1 showed cytoplasm and nucleus staining: strong cyclin D1 in Group 2, weaker staining of cyclin D1 in Group 3 and Group 4, and the weakest staining of cyclin D1 in Group 5 (Magnification 200×). Scale bars in the figures indicate 100 μm

**Fig. 6:**
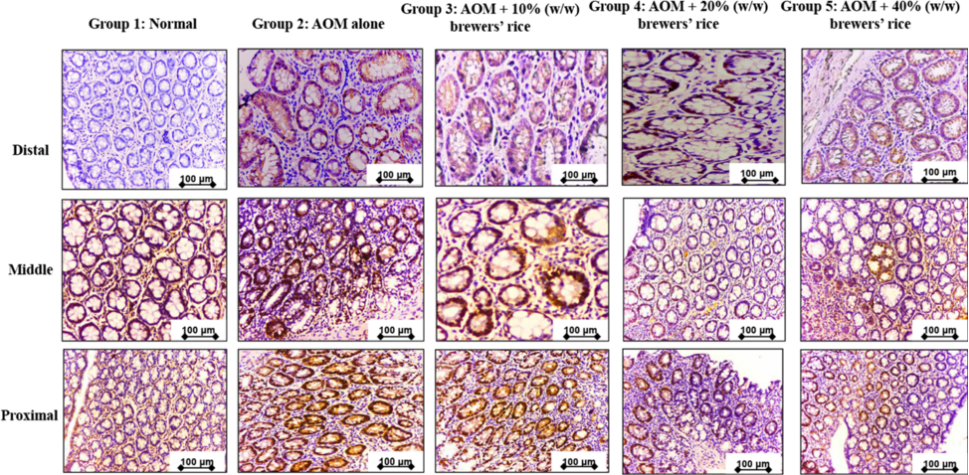
Immunohistochemical staining of c-myc. The expression of c-myc in three sections, including distal, middle, and proximal colon in normal (Group 1), AOM alone (untreated) (Group 2) compared to groups of rats treated with 10 % (Group 3), 20 % (Group 4), and 40 % (Group 5) (w/w) of brewers’ rice (*n* = 3). Expression of c-myc showed cytoplasm and nucleus staining: strong c-myc in Group 2, weaker staining of c-myc in Group 3 and Group 4, and the weakest staining of c-myc in Group 5 (Magnification 200×). Scale bars in the figures indicate 100 μm

Because cyclin D1 and c-myc are downstream signaling targets in Wnt signaling and the overexpression of cyclin D1 and c-myc are found in most patients with colorectal tumors, in whom lowering the expression of cyclin D1 has therapeutic significance [45], we investigated whether brewers’ rice suppresses the cyclin D1 and c-myc expression in colon tumors. The positive brownish staining of cyclin D1 and c-myc in the AOM-alone group and the brewers’ rice-administered groups were mostly localized in the cytoplasm and nucleus. The colon tumors from the AOM-alone group demonstrated intense staining for cyclin D1 and c-myc compared with those from the brewers’ rice-fed group. Reductions in the staining intensities for cyclin D1 and c-myc were also found in colon tumors from rats received with a diet containing brewers’ rice (Figs. 5 and 6).

Immunohistochemical analysis showed that the colon mucosa of AOM treated rats, which presented the highest values of tumor formation among all of the groups [16], demonstrated the highest score from the evaluation of the immunohistochemical analysis of cyclin D1 compared with the other treatment groups (Table 3). Consistent with the high expression of cyclin D1 in colon tumors, we also found the highest score of c-myc expression in the AOM-alone group compared with the other treatment groups (Table 3). The dietary administration of brewers’ rice has been demonstrated to diminish cyclin D1 expression in a dose-dependent manner. A similar trend was also observed in c-myc expression in AOM-injected rats administered brewers’ rice. The heterogeneous expression of cyclin D1 and c-myc was observed in the treatment groups (G3, G4, and G5), but only two treatment groups (20 and 40 % (w/w) of brewers’ rice) showed a significant reduction in cyclin D1 and c-myc expression compared with AOM alone (*P* < 0.05). However, no significant difference in both cyclin D1 and c-myc was found between the AOM alone and 10 % (w/w) brewers’ rice treatments (*P* > 0.05) (Table 3). These findings showed that brewers’ rice has the potential to decrease the downstream signaling targets cyclin D1 and c-myc. Overall, the data presented in this study suggest that brewers’ rice may modulate colon tumor development through Wnt/β-catenin signaling.

**Table 3:** Total score of cyclin D1 and c-myc expression in colonic tissue

Groups	Normal	AOM alone	AOM + 10 % (w/w) of brewers’ rice	AOM + 20 % (w/w) of brewers’ rice	AOM + 40 % (w/w) of brewers’ rice	P
Cyclin D1	0	123.67 ± 9.61^a^	123.00 ± 9.54^a^	94.33 ± 4.73^b^	85.67 ± 8.14^b^	0.000
c-myc	0	125.00 ± 5.57^a^	123.00 ± 8.00^a^	97.00 ± 4.58^b^	93.00 ± 6.08^b^	0.000

Each value expressed as mean ± SD of three determinations. Value in the same row with different superscript letter indicates significant difference by Tukey’s test (*P* < 0.05)

### Treatment with brewers’ rice reduces the protein level of β-catenin in colonic tumors

To investigate the inhibitory effects of brewers’ rice in regulating the Wnt signaling pathway, we employed AOM-induced colonic tumors, which are known to exert the wild-type β-catenin and truncated *APC* gene [46]. We hypothesized that brewers’ rice would downregulate the β-catenin, cyclin D1, and c-myc protein levels through the Wnt/β-catenin signaling pathway. Activation of the Wnt/β-catenin signaling pathway has been associated with colorectal tumors [47].

**Fig. 7:**
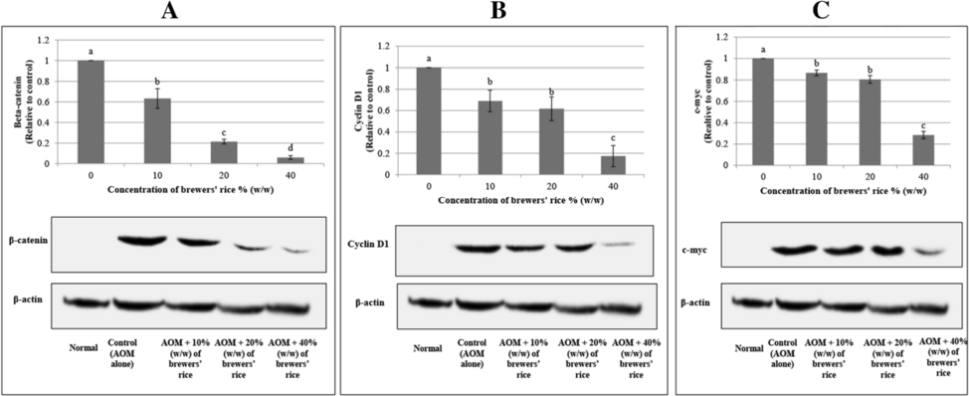
Western blotting of β-catenin, cyclin D1, and c-myc in brewers’ rice treated AOM-induced colon cancer. The protein levels of β-catenin, cyclin D1, and c-myc in normal (Group 1), AOM alone (untreated) (Group 2) compared to groups of rats treated with 10 % (Group 3), 20 % (Group 4), and 40 % (Group 5) (w/w) of brewers’ rice. **a** Brewers’ rice decreased protein expression of β-catenin, **b** brewers’ rice reduced protein expression of cyclin D1, and **c** brewers’ rice decreased protein expression of c-myc in a dose-dependent manner. Each value expressed as mean ± SD (*n* = 3). Value with different superscript letter indicates significant difference between groups by Tukey’s test (*P* < 0.05). Administration of 10, 20, and 40 % (w/w) of brewers’ rice significantly reduced β-catenin, cyclin D1, and c-myc protein expression compared to the AOM-alone group (*P* < 0.05). However, the protein levels of cyclin D1 and c-myc did not significantly differ between 10 % and 20 % (w/w) brewers’ rice (*P* > 0.05)

The protein level of β-catenin was diminished accordingly when the concentration of brewers’ rice was increased, indicating that brewers’ rice increased the degradation of β-catenin in a dose-dependent manner (Fig. 7a). Expectedly, we found no β-catenin protein expression in the normal colon tissue. This result may reveal that the β-catenin amount in the normal colon tissue was too low to be detected through western blotting. In the AOM-alone group (G2), a carcinogen was injected, but no treatment with brewers’ rice was administered, and prominent β-catenin protein expression was observed. The overall analysis showed that the colon mucosa of G2, which had the highest values of tumor incidence and tumor multiplicity compared with other groups [16], displayed the highest β-catenin protein expression in the western blotting evaluation compared with the other treatment groups.

A significant reduction in the protein expression of β-catenin was also found in the treatment groups (G3, G4, and G5) compared with G2 (*P* < 0.05) (Fig. 7a). This result was consistent with the findings obtained by Nurul Husna et al. [48], who reported that the protein level of β-catenin was diminished after treatment with rice bran phytic acid. These data implied that brewers’ rice has the potential to reduce the active form of β-catenin.

### Treatment with brewers’ rice reduces the cyclin D1 and c-myc protein levels in colonic tumors

To evaluate whether the dietary administration of brewers’ rice on AOM-injected colon carcinogenesis mediates the inhibition of the Wnt/β-catenin downstream signaling pathway, we investigated the Wnt target genes at the protein level. Cyclin D1 is a well-recognized cell cycle protein targeted by β-catenin [6] that is usually overexpressed in colonic tumors [49]. c-myc is one of the crucial protein in the modulation of cell proliferation by β-catenin and the Wnt pathway [50].

Expectedly, we observed no cyclin D1 and c-myc protein expression in the normal colon mucosa (Fig. 7b and c). As shown in Fig. 7b and c, the AOM-alone group (G2) exhibited the highest protein expression of cyclin D1 and c-myc in the western blotting analyses compared with the other treatment groups. We also found reductions in the cyclin D1 and c-myc protein levels in the colon tumors of rats received with a brewers’ rice diet. The dietary administration of brewers’ rice has been demonstrated to reduce cyclin D1 and c-myc protein expression in a dose-dependent manner. The protein expression of cyclin D1 and c-myc in the AOM-alone group was significantly higher than that of the brewers’ rice-treated groups (*P* < 0.05) (Fig. 7b and c). However, no significant difference in the cyclin D1 and c-myc protein expression levels was observed between the groups treated with 10 % and 20 % (w/w) brewers’ rice (*P* > 0.05). These results suggested that the effects of the dietary administration of brewers’ rice on cyclin D1 and c-myc corroborated the potential of brewers’ rice for regulating the β-catenin level in colon tumors. Taken together, this finding indicated that the dietary administration of brewers’ rice inhibited the Wnt/β-catenin downstream signaling pathway.

In the present study, the colon tumor samples in each group were randomly selected for analysis. Only three determinations conducted for each group in both immunohistochemistry and Western blotting analyses were based on several previous studies [48, 51–53].

Most studies have reported the additive and/or synergistic protective effects of several components [54]. Therefore, in the current study, brewers’ rice rather than isolated compounds were fed to the rats. In our previous study, we identified a greater inhibition of tumor incidence and multiplicity in AOM-induced rats after treatment with brewers’ rice [16]. We speculated that this was partially a result of the nutrient compositions and bioactive compounds, which synergistically contribute to this significant suppression of β-catenin, cyclin D1, and c-myc expression. Additionally, the synergistic/additive effects of the components and the presence of other active compounds in WBR such as phenolic compounds (mainly ferulic acid, gallic acid, and p-coumaric acid) [19], phytic acid, and antioxidant activity as evaluated using β-carotene bleaching test, 1,1-diphenyl-2-picryl-hydrazyl (DPPH) radical scavenging capacity, and ferric reducing antioxidant power (FRAP) assays [14] are more likely to cause the observed apoptotic effects in HT-29 cells, as confirmed by a colorimetric assay. A dietary chemoprevention study demonstrated that the magnitude of the anticancer effect in the whole food or whole food extract is greater compared to that of the individual phytochemical components [55]. Whole rice bran is recognized to be vital in providing a comprehensive protection against cancerous cells compared with the protection provided by the isolated compounds [56]. Therefore, the downregulation of the downstream signaling targets, including cyclin D1 and c-myc, are likely attributed to the synergistic/additive effects of the active constituents present in brewers’ rice, such as phenolic compounds (mainly ferulic, gallic, and p-coumaric acids), phytic acid, antioxidant activity, vitamin E (mainly γ-tocotrienol and γ-tocopherol), and γ-oryzanol, as reported in our previous studies [14, 19].

## Conclusions

This study provides clear and substantial evidence that brewers’ rice offers great potential against colorectal cancer through the inhibition of colon carcinogenesis by the modulation of upstream and downstream components in the Wnt signaling pathway. Our findings demonstrated that brewers’ rice downregulates β-catenin and the Wnt target proteins cyclin D1 and c-myc, which provides a good rationale for a further comprehensive study of dietary brewers’ rice against colon cancer. Collectively, these findings provide evidence that WBR can induce apoptosis and inhibit the proliferation of HT-29 cells through regulation of caspase-dependent pathways and suppression of the Wnt/β-catenin downstream signaling pathway in AOM-induced rats. We suggest that brewers’ rice may be a useful dietary agent for colorectal cancer.

## Abbreviations

AIN-93G: American institute of nutrition
AOM: Azoxymethane
APC: Adenomatous polyposis coli
CK1: Casein kinase 1
DMEM: Dulbecco’s modified eagle medium
DPPH: 1,1-diphenyl-2-picryl-hydrazyl
FBS: Fetal bovine serum
FRAP: Ferric reducing antioxidant power
Fz: Frizzled
GAPDH: Glyceraldehyde-3-phosphate dehydrogenase
GSK3β: Glycogen synthase kinase 3β
HRP: Horseradish Peroxidase
HT-29: Colorectal cancer
LDH: Lactate dehydrogenase
LEF: Lymphoid enhancing factor
LRP6: Low-density lipoprotein receptor-related protein 6
MBR: Methanol extract of brewers’ rice
MTT: 3-(4,5-dimethylthiazol-2-yl)-2,5-diphenyltetrazolium bromide
NSAIDs: Non-steroidal anti-inflammatory drugs
PBS: Phosphate-buffered saline
PBST: Phosphate-buffered saline in 0.1 % Tween-20
*p*NA: *p*nitroanilide
RIPA: Radio immune precipitation assay
SDS-PAGE: Sodium dodecyl sulfate-polyacrylamide gel electrophoresis
TCF: T-cell factor
WBR: Water extract of brewers’ rice
